# Desmoplastic Ameloblastoma: A case report of rare pulmonary metastasis

**DOI:** 10.1016/j.heliyon.2024.e40535

**Published:** 2024-11-21

**Authors:** Takaaki Kamatani, Ryogo Katada, Masataka Watanabe, Tatsuo Shirota

**Affiliations:** Department of Oral and Maxillofacial Surgery, Showa University School of Dentistry, Tokyo 145-8515, Japan

**Keywords:** Desmoplastic ameloblastoma, Pulmonary metastasis, Lung, Malignant ameloblastoma

## Abstract

Pulmonary metastasis of ameloblastoma is a rare associated with the histopathologically plexiform types of ameloblastoma. In this report, we present an exceptionally rare case of pulmonary metastatic ameloblastoma without local recurrence, emerging 12 years post-initial resection. A female patient, initially diagnosed with mandibular desmoplastic ameloblastoma, revealed masses in both lung fields of the lung on chest radiography, while chest computed tomography revealed more than 10 nodules in both lungs. Histopathological analysis of a lung biopsy specimen revealed a nest-like structure of tumor cells surrounded by highly columnar cells, exhibiting a palisade-like arrangement, and squamous metaplasia.

Subsequently, the diagnosis of pulmonary metastatic ameloblastoma was confirmed through immunohistochemical examination. Despite clinical trial treatment with molecular targeted therapy for three years, the lung disease remained clinically stable. This case highlights the need for oral surgeons to recognize that pulmonary metastasis of desmoplastic ameloblastoma can manifest as a late recurrence, extending beyond a decade after initial curative surgery.

## 1 Introduction

Ameloblastomas are common benign odontogenic tumors that typically arise near the mandibular angle. Despite their being nature, they frequently recur following surgical resection, with recurrences commonly observed in the maxilla and mandible. Desmoplastic ameloblastomas, distinguished by pronounced stromal desmoplasia, are distinct variants easily discernible clinically, radiographically, and histologically [1]. While local recurrence is relatively common, occurring in 50–72 % of cases post-surgery, distant metastasis of ameloblastoma is a rare phenomenon and typically follows a follicular histological pattern [2].

In this report, we present a unique case of pulmonary metastatic ameloblastoma that emerged 12 years after the initial resection, without local recurrence. The patient's initial diagnosis was desmoplastic ameloblastoma of the mandible. This occurrence challenges the conventional understanding of ameloblastoma behavior and underscores the importance of monitoring even long-term postoperative cases for potential metastatic events.

## 2 Case presentation

**Fig. 1:**
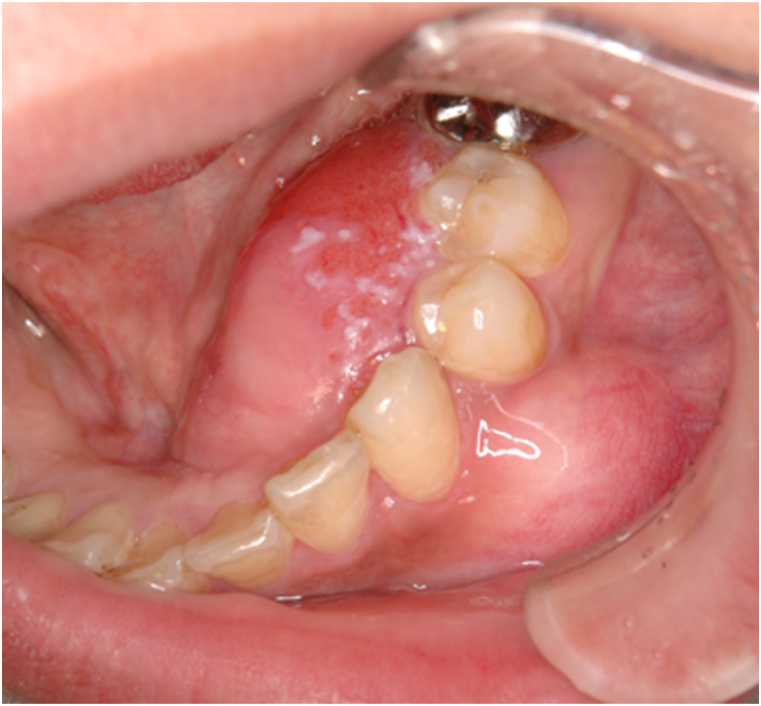
Intraoral view at first visit. A slow-growing mass is evident on the left side of the lingual and buccal lower gingiva.

**Fig. 2:**
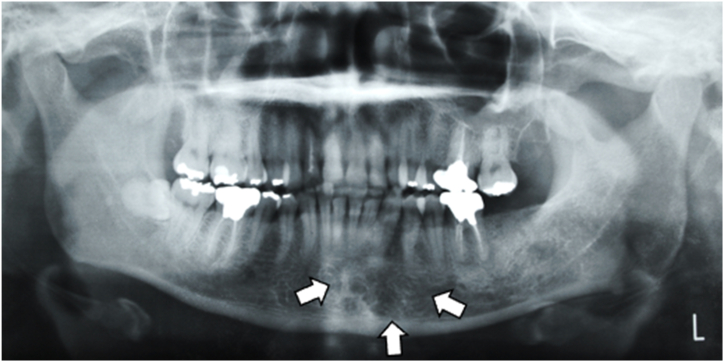
Orthopantomogram of the left side of the mandible. A mixed radiolucent–radiopaque lesion is visible.

**Fig. 3:**
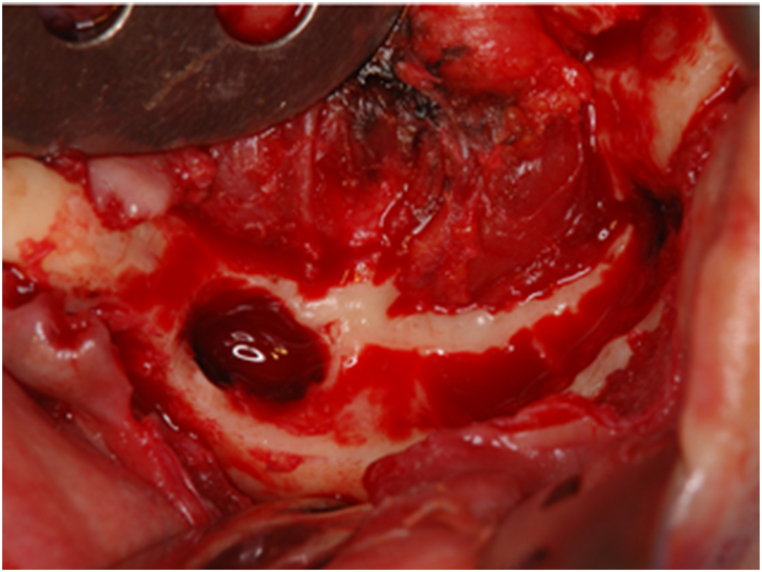
Surgical photographs. An intraoperative photograph are shown.

**Fig. 4:**
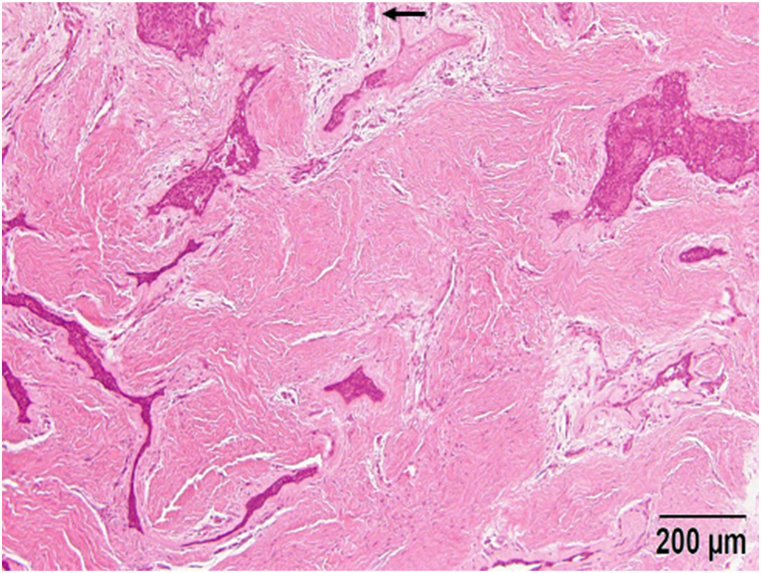
Histological findings of the tumor. An irregularly shaped epithelial island is shown surrounded by narrow zones of loose-structured connective tissue embedded in the desmoplastic stroma.

A 43- year-old female patient presented to our department with a swelling on the left side of the mandible that developed gradually over more than 10-years. Intraoral examination revealed an ill-defined solitary swelling on the left side of the premolar buccal gingiva, accompanied by slight redness and swelling on the left side of the premolar lingual gingiva, measuring approximately 4.0 × 3.0 cm (Fig. 1). Her medical history included hypertension, primary aldosteronism, and a uterine myoma. Radiographic examination of the mandible showed a diffuse, ill-defined, mixed radiolucent, and radiopaque lesion extending from the central incisor to the second premolar, approximately 3.0 × 2.5 cm (Fig. 2). Based on the clinical and radiographic findings, a preliminary diagnosis of a benign tumor on the left side of the mandible was made. Subsequently, a partial mandibulectomy was performed under general anesthesia to resect the tumor (Fig. 3). Histological examination revealed epithelial islands and collagenous stroma, leading to the final diagnosis of desmoplastic ameloblastoma of the mandible (Fig. 4).

**Fig. 5:**
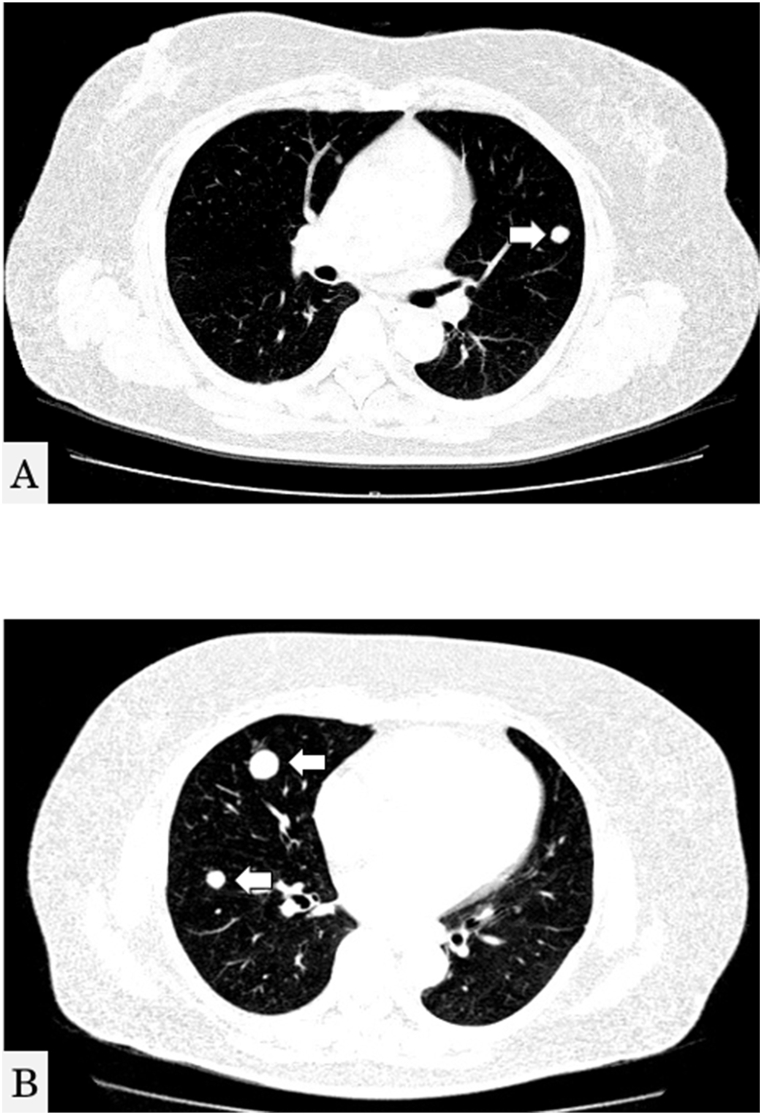
Chest computed axial tomography scan. Multiple well-defined nodules in both lungs are shown.

**Fig. 6:**
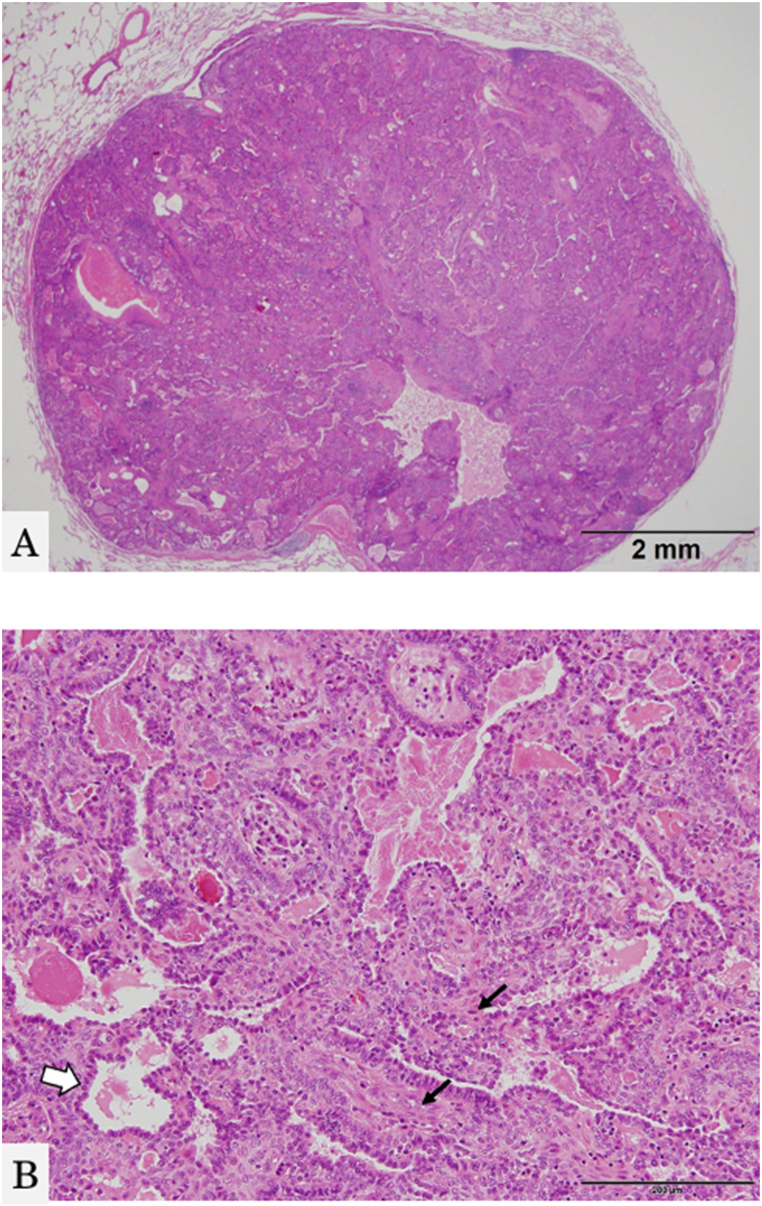
Histopathological findings and immunohistochemical results from a biopsy specimen of the lung tumor. The pulmonary nodule shows a nest-like structure of tumor cells, and the surrounding cells exhibit a high columnar shape, a palisade-like arrangement, and squamous metaplasia. A: HE, low-power view, B: HE, high-power view.

Despite the absence of respiratory symptoms, including occasional or recurrent cough, hemoptysis, dyspnea, or decreased blood oxygen saturation, a mass in both lung fields was detected by chest radiography during a medical health checkup 12 years post-operation. No abnormalities or recurrences were observed on the left side of the mandible. Chest computed tomography (CT) revealed more than 10 nodules in both lungs (Fig. 5A and B). Biopsy of the peripheral pulmonary lesions using bronchoscopy revealed a nest-like structure of tumor cells, with surrounding cells exhibiting a highly columnar shape, a palisade-like arrangement, and squamous metaplasia (Fig. 6A and B). Immunohistochemical examination results were positive for cytokeratin (CK) 7, 19, and 5/6, thyroid transcription factor-1 (TTF-1), p40, and calretinin. These findings suggested that the lung tumor did not originate from the lung tissues, as seen in lung adenocarcinoma, squamous cell carcinoma, or small-cell lung cancer. The final diagnosis was pulmonary metastatic ameloblastoma, originally diagnosed as desmoplastic ameloblastoma. As the patient is *BRAF* V600E-positive, she is undergoing a clinical trial of molecular targeted therapy.

## 3 Discussion

Metastatic ameloblastomas are rare, with fewer than 2 % of ameloblastoma cases showing distant metastasis, primarily to the lungs. Despite aggressive surgical management, prognosis remains poor [3]. Metastatic ameloblastomas displays a range of histopathological features. In 2005, the World Health Organization established guidelines to differentiate ameloblastoma from malignant ameloblastoma and ameloblastic carcinoma [4,5]. Malignant ameloblastomas, through histologically benign, exhibit metastasis with characteristics mirroring the primary oral tumors. On the contrary, ameloblastic carcinoma combines features of a typical ameloblastoma but showcases cytological atypia or an anaplastic appearance with or without metastasis [5]. In our case, the absence of cellular features of malignancy in both the tumor mass and lung metastasis suggested classification as malignant ameloblastoma rather than ameloblastic carcinoma. Notably, only one case of desmoplastic ameloblastoma metastasis has been reported in the English literature [6].

The 12-year interval between surgical resection and pulmonary metastasis in our patient may appear unusual; however, distant metastasis of ameloblastoma has been documented in the lungs over a decade after the primary operation, followed by metastasis to cervical lymph nodes, bones, brain, liver, spleen, kidney, diaphragm, and heart [7]. Lung metastasis commonly occurs bilaterally, with a reported rate of less than 2 % [8]. The proposed mechanisms of metastatic ameloblastoma spread include hematogenous and lymphatic routes, possibly initiated during surgical procedures or aspiration of tumor cells from the primary oral lesion [8]. Given its bilateral occurrence in the lungs and presentation with multiple nodules, hematogenous spread aligns with the theory of metastatic ameloblastoma [4]. Typically, the development of metastatic tumors occurs over an average of 10–12 years after the primary tumor occurrences [9]. Oral surgeons should be vigilant, recognizing that pulmonary metastasis of desmoplastic ameloblastoma may manifest more than a decade after curative primary surgery. Annual chest radiography is recommended for follow-up after radical surgical resection of ameloblastoma.

The primary site for metastasizing ameloblastoma is typically the mandibular ameloblastoma. Metastasizing ameloblastomas may manifest bilaterally in the lungs, with a mean interval of 18 years after surgery [4]. Previous studies have documented metastases to the lungs, liver, and kidneys [4,10,11]. In our patient, potential metastatic pathways included hematogenous or lymphatic routes, or inflow from the trachea to the lungs, as mentioned earlier [12].

Treatment for metastasizing ameloblastoma varies, including options such as surgical resection, chemotherapy, and radiotherapy [13]. In some cases, no treatment is pursued due to slow tumor cell proliferation in metastatic foci, although patient numbers are limited [12]. Recent studies have identified the *BRAF* V600E mutation in 63 % of ameloblastoma lesions, leading to the use of the molecular-targeted drug dabrafenib. This drug, employed for *BRAF* gene mutation-positive cancers, has shown promising results in reducing or eliminating tumors [14]. In our patient, radical surgery posed challenges, leading to participation in a clinical trial for molecular target therapy.

While primary management of malignant ameloblastoma is effectively addresses oral lesions, there is insufficient information regarding the treatment of metastatic ameloblastoma without local recurrence. En-bloc resection, with a 10- to 15-mm safety margin of normal bone, is common during tumor removal. Proposed treatments for metastatic ameloblastoma include close observation, thoracotomy with wedge resection, or experimental chemotherapy [4]. Surgical resection may be challenging in some cases due to anatomical location or tumor extension. This case report, along with future studies, is pivotal for developing comprehensive treatment guidelines for patients with metastatic ameloblastoma.

## 4 Conclusion

This case report contributes to the evolving understanding of metastatic ameloblastomas and underscores the complexities involved in their diagnosis and treatment. Continued research, documentation of cases, and exploration of targeted therapies are essential for developing comprehensive guidelines to address the unique challenges posed by metastatic ameloblastomas.

## CRediT authorship contribution statement

**Takaaki Kamatani:** Writing – original draft, Project administration, Conceptualization. **Ryogo Katada:** Data curation, Writing – original draft. **Masataka Watanabe:** Visualization, Writing – review & editing. **Tatsuo Shirota:** Conceptualization, Data curation, Formal analysis.

## Ethics approval and consent to participate

Written informed consent was obtained from the patient for participation in this case report and any accompanying images.

## Consent for publication

Not applicable.

## Availability of data

All data to support the conclusion have been provided in this article.

### Funding

This study received no specific grant from any funding agency.

## Declaration of competing interest

The authors declare that they have no known competing financial interests or personal relationships that could have appeared to influence the work reported in this paper.
